# Ab initio protein structure prediction: the necessary presence of external force field as it is delivered by Hsp40 chaperone

**DOI:** 10.1186/s12859-023-05545-0

**Published:** 2023-11-07

**Authors:** Irena Roterman, Katarzyna Stapor, Leszek Konieczny

**Affiliations:** 1 Department of Bioinformatics and Telemedicine, Jagiellonian University - Medical College, Medyczna 7, 30-688 Krakow, Poland; 2 Department of Applied Informatics, Silesian University of Technology, Gliwice, Poland; 3 Chair of Medical Biochemistry, Jagiellonian University - Medical College, Kopernika 7, 31-034 Krakow, Poland

**Keywords:** Chaperone, Hsp40, DnaJ, Alkaline phosphatase, Folding, Client molecule

## Abstract

**Background:**

The aqueous environment directs the protein folding process towards the generation of micelle-type structures, which results in the exposure of hydrophilic residues on the surface (polarity) and the concentration of hydrophobic residues in the center (hydrophobic core). Obtaining a structure without a hydrophobic core requires a different type of external force field than those generated by a water. The examples are membrane proteins, where the distribution of hydrophobicity is opposite to that of water-soluble proteins. Apart from these two extreme examples, the process of protein folding can be directed by chaperones, resulting in a structure devoid of a hydrophobic core.

**Results:**

The current work presents such example: DnaJ Hsp40 in complex with alkaline phosphatase PhoA-U (PDB ID—6PSI)—the client molecule. The availability of WT form of the folding protein—alkaline phosphatase (PDB ID—1EW8) enables a comparative analysis of the structures: at the stage of interaction with the chaperone and the final, folded structure of this biologically active protein. The fuzzy oil drop model in its modified FOD-M version was used in this analysis, taking into account the influence of an external force field, in this case coming from a chaperone.

**Conclusions:**

The FOD-M model identifies the external force field introduced by chaperon influencing the folding proces. The identified specific external force field can be applied in Ab Initio protein structure prediction as the environmental conditioning the folding proces.

## Introduction

Chaperone proteins play a significant role in a control of protein folding process [1]. Their function relies on preventing the formation of internal interactions unfavorable for the final structure of proteins. The risk of misfolding increases when the temperature increases. The heat shock proteins are a group of chaperone proteins, the production of which is increased under conditions of thermal stress [2]. Based on the size of the chaperon molecule, the following classes are distinguished: Hsp90, Hsp70 and Hsp40, where the number at the end is the number of kilodaltons of a given group of heat shock proteins. The most frequently identified proteins in the Hsp group are Hsp70 and Hsp40, also referred to as DnaK and DnaJ, respectively [3]. The structures of proteins supporting the folding process, including the prevention of inappropriate aggregation, as well as the potential interaction with the membrane, are the subject of numerous studies, as the mechanism of action of these proteins is not fully recognized [4, 5]. In order for chaperone proteins to perform their functions, it is necessary to identify the sites of chaperone-client interactions [6, 7]. Very often, in the activity of chaperone proteins, a specific synergy involving both Hsp40 and Hsp70 is observed [8–10].

The availability of chaperone’s structure together with the structure of the folding protein in a complex is a valuable source of information on the relationships between these two types of proteins during the folding process. The object of analysis in the current work is the complex of two Hsp40 chaperone chains (ttHsp40—tt-*Thermus thermophilus*) with a pre-folded Alkaline Phosphatase (PhoA) chain. This complex (PDB ID 6PSI—[11]) and its components (two chaperone chains and a partially folded "client" protein) are analyzed individually as independent structural units. For comparative purposes, the final structure of the folded client protein (PDB ID 1EW8—[12]) is also used in this work.

The aim of the current work is to quantify the impact of different environmental factors, including the chaperone’s one, on the folding process of the aforementioned client protein. The paper presents a proposal for expressing the external force field for the representation of environmental conditions for folding proces in ab initio approach. This proposal relies on the assumption that a polypeptide chain built-up of bi-polar amino acids with different proportions of polar to hydrophobic parts in a polar water environment tends to obtain a micelle-like structure. This means the concentration of hydrophobic residues in the center of the molecule with the exposure of polar ones on the surface. The hydrophobicity distribution resulting from such idealized structuring can be described by 3DGaussian function. This function is proposed to represent the external force field directing the folding proces. Real proteins, each with a specific sequence can obtain a micelle-like structure to a full or limited extent which can be expressed by a local mismatch of the observed hydrophobicity distribution to the idealized one. The status of the hydrophobicity distribution present in a given structure is determined on the basis of the hydrophobic interactions as dependent on the distances between the interacting residues and their different intrinsic hydrophobicities.

A specific form and degree of maladjustment of the hydrophobicity distribution in the protein in relation to the idealized one is a form of recording the specific activity of a given protein. Local maladjustment carries information about the specificity of a potential partner in a chemical reaction. In the case of enzymes, the local "deficit" of hydrophobicity expresses the presence of cavity ready to interact with the ligand/substrate. Local exposure ("excess") of hydrophobicity on the surface may in turn be the form of preparation to complexation of another protein [13].

Just as the external environment provides the conditions for proper folding, the already folded protein can provide a local external force field for the course of a given reaction in the active site of the enzyme. The protein body is much larger than the active center, constituting an external force field for a specific reaction. The degree and type of separateness of the hydrophobicity distribution from the idealized one is a kind of recording the specificity of the environment and its impact on the ongoing process in the active center. The structure of the protein, including its local maladjustment to the micelle-like system, is the result of the influence of the external environment. The order/disorder of the hydrophobicity distribution in the folded protein, in turn, constitutes an external force field for the active site and thus an environment for a specific reaction.

In the Fuzzy oil drop (FOD) model [14], the external force field for proteins folding in an aqueous environment is described by the 3DGauss function. Proteins with an ordered hydrophobicity distribution in this way are known [15]. A different specificity is expressed by the membrane environment in which numerous proteins are active. The modification of the FOD model was introduced in the FOD-M model [15], where the possibility of changing the characteristics of the environment is taken into account. In addition to the factors such as the cell membrane, other factors are used to guide the process of protein folding. Such factors are chaperones, which—as it is generally interpreted—prevent misfolding by imposing the appropriate structuring.

In the present study the assessment of the form and degree of influence of the chaperone on the protein folding process was carried out by analyzing the native form of bacterial alkaline phosphatase [12] with the form that this protein represents in a complex with the chaperone Hsp40—DnaJ2 [11]. The available structures, both native and partially folded with the help of a chaperone, enables the comparative analysis to track the structural changes during folding.

It is also possible to identify the specificity of an external force field provided by a chaperon. The status of the hydrophobicity distribution in the native form revealing significant deviations from the distribution typical for the aquatic environment allows to determine the influence of the external force field introduced by a chaperone. In turn. the availability of the structure of the chaperone-client molecule’s complex, enables the evaluation of the chaperone itself as a provider of a specific external force field for the folding of the enzyme in question.

The FOD-M model is assumed to deliver the expression of environmental conditions directing the folding process toward the aim-oriented results in biologicaly active form.

**Fig. 1:**
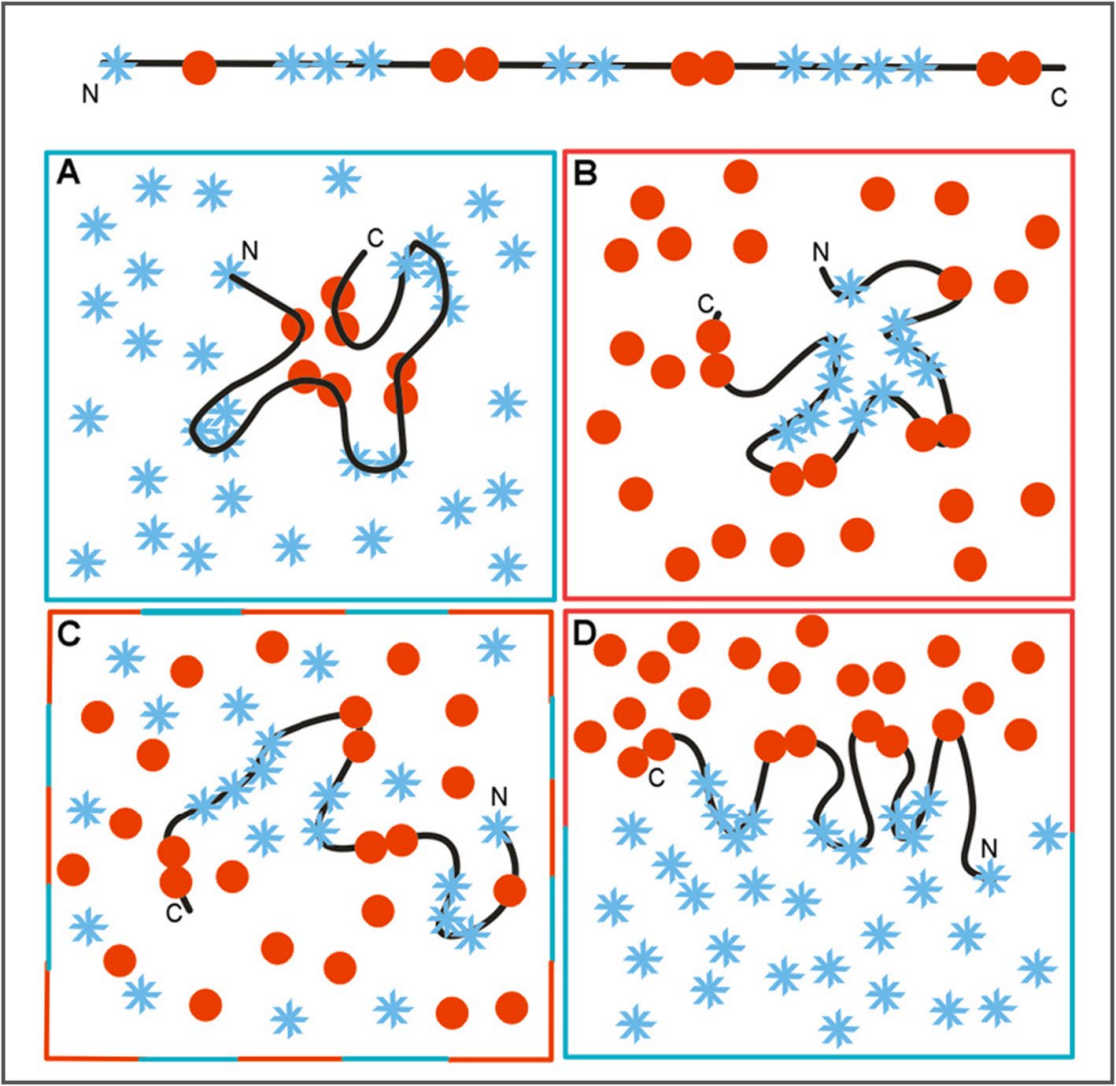
Different structuralisations of polypeptide chain of the sequence of polar (blue stars) and hydrophobic residues (red circles) (top line) depending on othe form of external force field. **A** Water environment directs the folding toward the micelle-like structuralisation with hydrophobic core and polar surface. **B** Hydrophobic environment directs the folding toward exposure of hydrophobic residues on the surface with polar residues concentrated in central part (membrane protein case). **C** Mixture of polar/hydrophobic compounds present in environment produces the unfolded structural form of polypeptide chain. **D** Bi-layer environment with separated polar and hydrophobic compounds directs folding process toward the polar structure with two poles: polar one and hydrophobic one

Pictorial presentation of the discussed model is shown in Fig. 1. The influence of polar water environment (Fig. 1A) supports the process of micellisation. The hydrophobic environment (similar to membrane environment) directs the structuralisation process toward polar core in central part of protein with hydrophobic residues exposed on the surface (Fig. 1B). The mixture of polar-water and hydrophobic compounds in random organisation produce the unflded form of polypeptide chain (Fig. 1C). The dynamic changes of polar/hydrophobic compounds positions causes the dynamic structural forms of polypeptide chain structuralisation. This example (Fig. 1C) represents the status of unfolded polypeptide chain. The ordered organisation of polar and hydrophobic in bi-layer form directs the folding process toward the di-polar organisation of the protein structure (Fig. 1D). All examples: Fig. 1B–D represent the structures deprived of hydrophobic core.

**Fig. 2:**
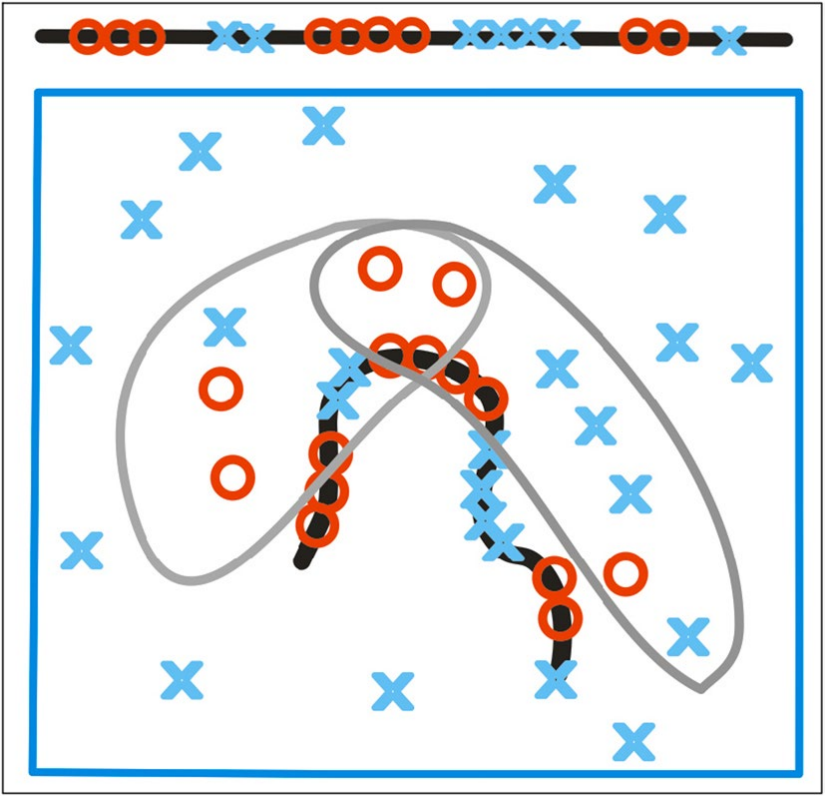
The chaperone (two gray parts) influence on folding process. The localisation of polar (blue X) and hydrophobic (red O) residues in polypeptide chain (client molecule) determined by positions of polar and hydrophobic residues in chaperone (external force field)

The presence of chaperone (discussed in this paper) plays also the role of external force field directing the folding process toward structure with hydrophobicity distribution as directed by external force field coded in chaperone (Fig. 2.).

The stability of polar and hydrophobic residues dispertion in chaperone (Fig. 2) produces the stable structralisation of polypeptide chain in contrast to example (Fig. 1C) where the localisation of polar and hydrophobic compounds is dynamic.

## Materials and methods

### Data

Table 1 shows the proteins analyzed with the identifiers introduced for the present work.

**Table 1:** Proteins with structural units analyzed in the work and identifiers introduced for the purpose of this work

PDB—ID	SKŁAD	NAME	ID in this paper
6PSI [11]	Chain A—chaperone	Hsp40-A	HspA-Comp (complex)
	Chain C—chaperone	Hsp40-C	
	Chain B—client	PhoA	
6PSI	Chain A—chaperone	Hsp40-A	HspA-AC
	Chain C—chaperone	Hsp40-C	
6PSI	Chain A—chaperone	Hsp40-A	Hsp40-A
6PSI	Chain C—chaperone	Hsp40-C	Hsp40-C
6PSI	Chain B—client	PhoA	PhoA-U (unfolded)
1EW8 [12]	Chain A	PhoA-A	PhoA-WT-AB
	Chain B	PhoA-B	
1EW8	Chain A	PhoA	PhoA-WT-A

The right column gives the identification system for structural units as described in this paper

### Programs used

The program allowing calculation of RD (Relative Distance—see Methods) is accessible upon request on CodeOcean platform: https://codeocean.com/capsule/3084411/tree. Please contact the corresponding author to get access to your private program instance (accessed June 10, 2023).

The application—implemented in collaboration with the Sano Centre for Computational Medicine (https://sano.science) and running on resources contributed by ACC Cyfronet AGH (https://www.cyfronet.pl) in the framework of the PL-Grid Infrastructure (https://plgrid.pl)—provides a web wrapper for the abovementioned computational component and is freely available at https://hphob.sano.science (accessed June 10, 2023).

The VMD program was used to present the 3D structures [16, 17] (accessed June 10, 2023).

### The FOD-M model

The FOD model assumes that the structure of proteins folding in a polar water environment leads to the formation of forms corresponding to a spherical micelle with a hydrophobic, centrally located hydrophobic core and a polar surface that guarantees a favorable entropic-enthalpy state towards the water environment. The description of the hydrophobicity distribution in the micelle-like form is expressed by the 3DGaussian function spanned over a protein molecule. The size of the ellipsoid (expressed by the appropriate values of σ_X_, σ_Y_ and σ_Z_) is adjusted to the size of the protein molecule encapsulating it.

1 $$H_{i}^{T} = \frac{1}{{H_{{sum}}^{T} }}\exp \left( {\frac{{ - \left( {x_{i} - \bar{x}} \right)^{2} }}{{2\sigma _{x}^{2} }}} \right)\exp \left( {\frac{{ - \left( {y_{i} - \bar{y}} \right)^{2} }}{{2\sigma _{y}^{2} }}} \right)\exp \left( {\frac{{ - \left( {z_{i} - \bar{z}} \right)^{2} }}{{2\sigma _{z}^{2} }}} \right).$$

**Fig. 3:**
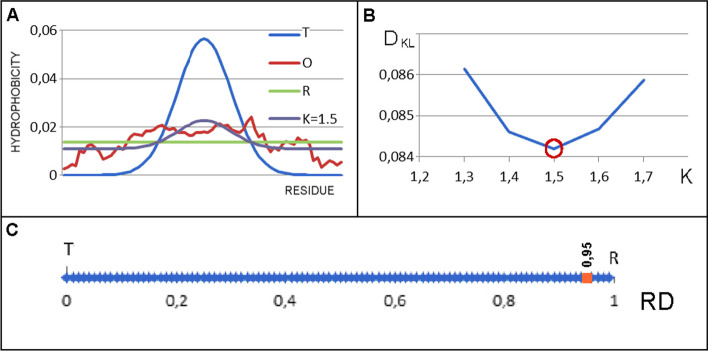
Graphical interpretation of the FOD and FOD-M models. **A** Profiles: T (blue), O (red), R (gray) and M (yellow) for K = 1.5. **B** Search for optimal K value—the minimum value of D_KL_ for (O|M) relations is reached for K = 1.5 in this case. **C** The RD value for O profile assesment—RD = 0.95 is obtained for the O distribution as similar to R distribution in this example

The value of the 3DGaussian function for the positions of effective atoms (average position of atoms included in a given amino acid) expresses the level of hydrophobicity treated as theoretical, completely consistent with the micelle-like system, and is denoted by T_i_ (Fig. 3A).

The actual level of hydrophobicity resulting from interactions is determined using the function proposed by Levitt [18]. The observed level of hydrophobicity assigned to a given effective atom—referred to as O_i_—depends on the distance between adjacent effective atoms (cutoff distance = 9 Å) and the intrinsic hydrophobicity of each interacting amino acid.

2 $$H_{i}^{O} = \frac{1}{{H_{{sum}}^{O} }}\sum\limits_{j} {\left\{ {\begin{array}{*{20}c} {\left( {H_{i}^{r} + H_{j}^{r} } \right)\left( {1 - \frac{1}{2}\left( {7\left( {\frac{{r_{{ij}} }}{c}} \right)^{2} - 9\left( {\frac{{r_{{ij}} }}{c}} \right)^{4} + 5\left( {\frac{{r_{{ij}} }}{c}} \right)^{6} - \left( {\frac{{r_{{ij}} }}{c}} \right)^{8} } \right)} \right)} & {for\;r_{{ij}} \le c} \\ {0,} & {for\;r_{{ij}} > c} \\ \end{array} } \right.}$$

Both T and O distributions after normalization can be compared by evaluating to what extent the O distribution reflects the T one. In other words, how close is the O distribution to the distribution of an idealized spherical micelle (Fig. 3A).

In order to quantify this distance, the D_KL_ entropy divergence introduced by Kullback–Leibler [19] was used. The D_KL_ value expresses the amount of entropy for the assessment of the differences between the two compared distributions P_i_ (the analyzed distribution—in our case the O distribution) and Q_i_—the reference distribution (in our case the T distribution)

3 $$D_{KL} \left( {P{|}Q} \right) = \mathop \sum \limits_{i = 1}^{N} P_{i} {\text{log}}_{2} \frac{{P_{i} }}{{Q_{i} }}.$$

The value of D_KL_ determined in this way cannot be interpreted as expressing entropy. Thus, a second reference distribution was introduced in the form of the R distribution, where each residue is described by the same level of hydrophobicity equal to R_i_ = 1/N where N is the number of amino acids in the protein. Such a distribution represents a status devoid of any variation in hydrophobicity levels within the protein and thus devoid of a hydrophobic core.

Each protein is described by the two parameters: D_KL_ for the O|T and for the O|R relationships, respectively. To avoid the necessity of using two parameters to describe one object, the RD (Relative Distance) parameter was introduced, expressed as:

4 $$RD = \frac{{D_{KL} (O|T)}}{{D_{KL} (O|T) + D_{KL} (O|R)}}$$

The value of RD < 0.5 means the presence of a hydrophobic core (good approximation of the O distribution the T one), while RD > 0.5 means the absence of a hydrophobic core (the O distribution similar to uniform R distribution) (Fig. 3C).

The RD parameter can be determined for any given structural unit: complex, single chain or domain. In this case, the corresponding 3DGaussian function is defined for each of the listed structural units.

It is also possible to assess the contribution to the construction of a given distribution of any selected section (or other component, e.g. a single protein within a complex, or a domain within a protein, but also a chain fragment within a given structural unit). For this purpose, the Oi and Ti values of the selected fragment are normalized and the calculation of the D_KL_s and RD values becomes possible. Interpretation of the RD value is as above: RD < 0.5 means the participation of a given fragment in the construction of a central hydrophobic nucleus. An RD value > 0.5 for a given fragment means a local disorder of the structure of the micelle-like system.

The water environment is not the only one where proteins are active. In particular, membrane proteins function in an environment that expects a different distribution of hydrophobicity. The hydrophobicity is expected to be exposed on the surface for stabilization in the hydrophobic environment of the membrane. Therefore, the idealized hydrophobicity distribution in a membrane protein is described by a function that is the complement of the 3DGaussian function:

5 $$M_{i} = T_{MAX} - T_{i}$$

On the basis of previous analyses, the description of the distribution of hydrophobicity in the membrane protein is a combination of the distribution of 3DGauss and [1-3DGauss], with the share of the latter turning out to be varied. Therefore, the final form of the hydrophobicity distribution turns out to be:

6 $$M_{i} = \left[ {T_{i} + K*\left( {T_{MAX} - T_{i} } \right)_{n} } \right]_{n}$$

where the index n means a normalization. Parameter K determines the degree to which the micelle-like system has been modified by the external factors, including the hydrophobicity coming from an environment in particular. The choice of the values of parameter K is determined on the basis of the minimum D_KL_ value calculated for the O|M relation, which means that the given modified M distribution was „reproducer” to the highest degree by the O distribution (Fig. 3B).

Therefore, each protein analyzed here is determined by two parameters: RD – the assessment of proximity to/far from the idealized micelle-like distribution and K, which determines the degree of participation of environmental factors other than polar water affecting the structure of the protein in question. Thus, the RD value characterizes the structuring of the protein, while the K parameter characterizes the environment.

Proteins with a high degree of micelle-like order with low R and K values have been identified. Proteins characterized by the K = 0 parameter are: down-hill, fast-folding, and antifreeze type II proteins. Also, the vast majority of domains present in proteins show structures with a micelle-like order of hydrophobicity [15]. Membrane proteins including channels in particular show significantly elevated RD and K values (even K levels as high as 3) [20, 21].

The analysis of protein complexation models showing compliance with the FOD model, based on the use of superficial hydrophobic interactions to build the interface, is a point of reference for the example analyzed in the present work. In [22] it has been shown that if the purpose of the complex is only to stabilize and maintain the system, then the construction of an interface is based on a common hydrophobic core for the interacting proteins. The interface is built of hydrophobic residues of interacting proteins exposed on the monomers surface. A local deviation of the O distribution from the T one in the form of a local excess of hydrophobicity indicates a potential site of complexation of another. A local hydrophobicity deficit indicates the presence of a cavity. The type of incompatibility determines the specificity towards a potential interaction partner. This is clearly seen in a significant proportion of enzymes [23].

The main aim of the present study is to characterize the chaperone protein DnaJ Hsp40 in a complex with the protein referred to as the "client" using FOD-M model as well as the folded client protein In its native form.

The example shown in Fig. 1A represents the status of low RD and low K as similar to 3D Gauss distribution. The examples shown in Fig. 1B–D as well as in Fig. 2 represent the status described by high RD and high K values. The aim of this paper is to assess the influence of chaperone on folding process. This influence is expressed by calculated values of RD and K parameters as they are present in the protein folded in assistance of chaperone.

## Results

The description of the results obtained in the current work covers the following issues.

1. The non-micelle-like structuring in PhoA-WT-Aprotein.
2. Tracking structural changes during PhoA-U folding into PhoA-WT-A.
3. Determining the specifics of the external force field introduced by the chaperone –HspA-Comp and HspA-AC
4. Speculations on how the chaperone chain obtains the structure – HspA-A

### Comparative analysis of PhoA-U and PhoA-WT structures: assessment of changes in the hydrophobicity distribution

Evaluation of the homodimer status of the active form of PhoA-AB reveals a high degree of maladjustment of the observed distribution to micelle-like one. A very high value of the K parameter indicates a significant contribution of environmental modifying factors to the polar water environment (Table 2). The status of PhoA-A and PhoA-B chains treated as components of the complex turns out to be comparable to the status of a complete complex with equally high K values.

**Table 2:** The set of parameters describing the status of the chains in the dimer and the monomers in the PhoA-WT structure

1EW8	Complex		Individual	
PhoA-WT	PhoA-WT-A / PhoA-WT-B		PhoA-WT-A / PhoA-WT-B // PhoA-U	
	RD	K	RD	K
PhoA-AB	0.689	1.1		
PhoA-A/PhoA-B	0.691 / 0.688	1.0 / 1.1	0.596 /0.596 // 0.778	0.6 / 0.6 // 1.7
CAT 102 ± 5	0.485 / 0.487		0.274 / 0.289 // 0.564	
CAT 166 ± 5	0.610 / 0.605		0.539 / 0.537 // 0.518	
SS 168–178	0.471 / 0.471		0.484 / 0.483 // 0.521	
SS 286–336	0.572 / 0.597		0.480 /0.476 // 0.592	
P-P	0.678		0.718 / 0.720	
NO P-P	0.672		0.574 / 0.577	

Two values given in the COMPLEX column—the status of the chains A and B considered as components of PhoA-WT. The columns COMPLEX/ INDIVIDUAL—3D Gauss function stretched over a complex/chain. Values after the // sign—status in the form of PhoA-U. CAT ± 5 denotes the status of the catalytic residue along with a stretch of immediate surroundings ± 5 adjacent residues. SS—segments defined by Cyx positions building the appropriate disulfide bond. P-P—the status of the residues included in the interface, NoP-P—the status of the rest of the chain after elimination of the interface residues

**Fig. 4:**
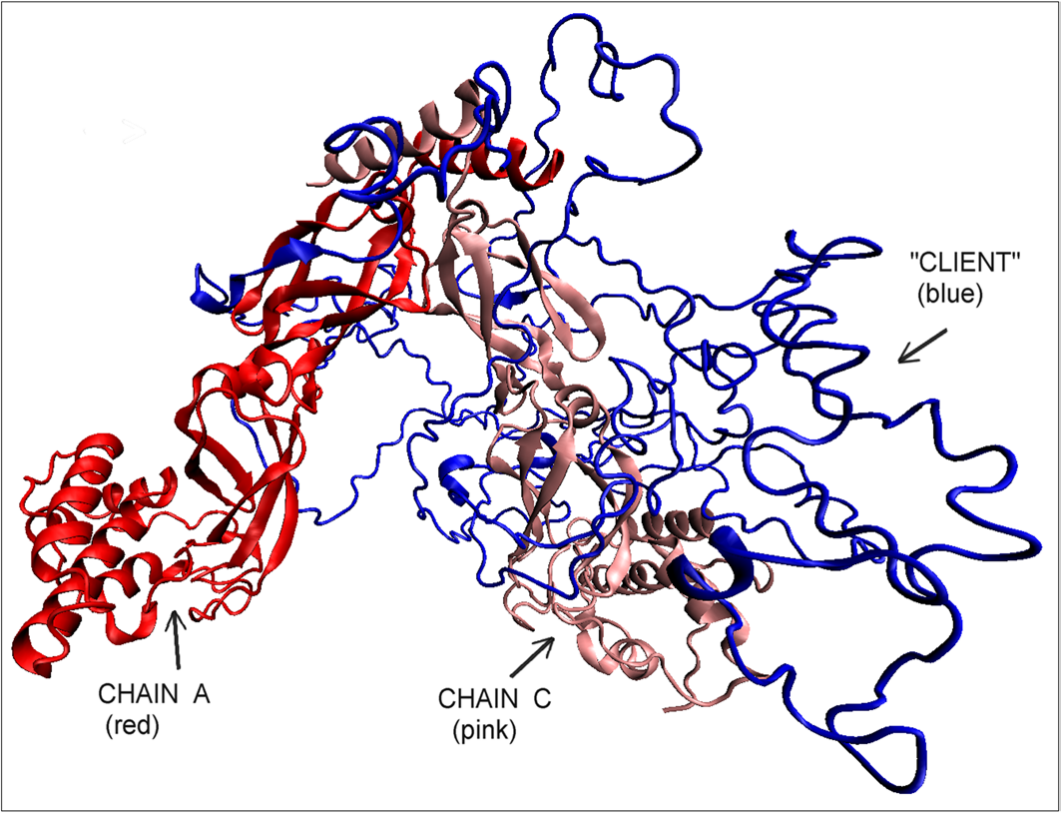
3D structure of the complete complex: Hsp40-A— red, Hsp40-C—pink, PhoA-U—"client” (chain B)—blue

PhoA-A and PhoA-B chains treated as individual units show slightly lower RD and K values, although they are far from those typical of micelle-like ones. On the other hand, the status of the PhoA-U chain is described by very high RD and K values—much higher than for the PhoA-WT. This is due to the very low packing of the structure caused by the cleavage of the PhoA-U chain between Hsp40-A and Hsp40-C (Fig. 4). It should be noted that the value of K = 1.7 is one of the highest values observed for polypeptide chain structures.

**Fig. 5:**
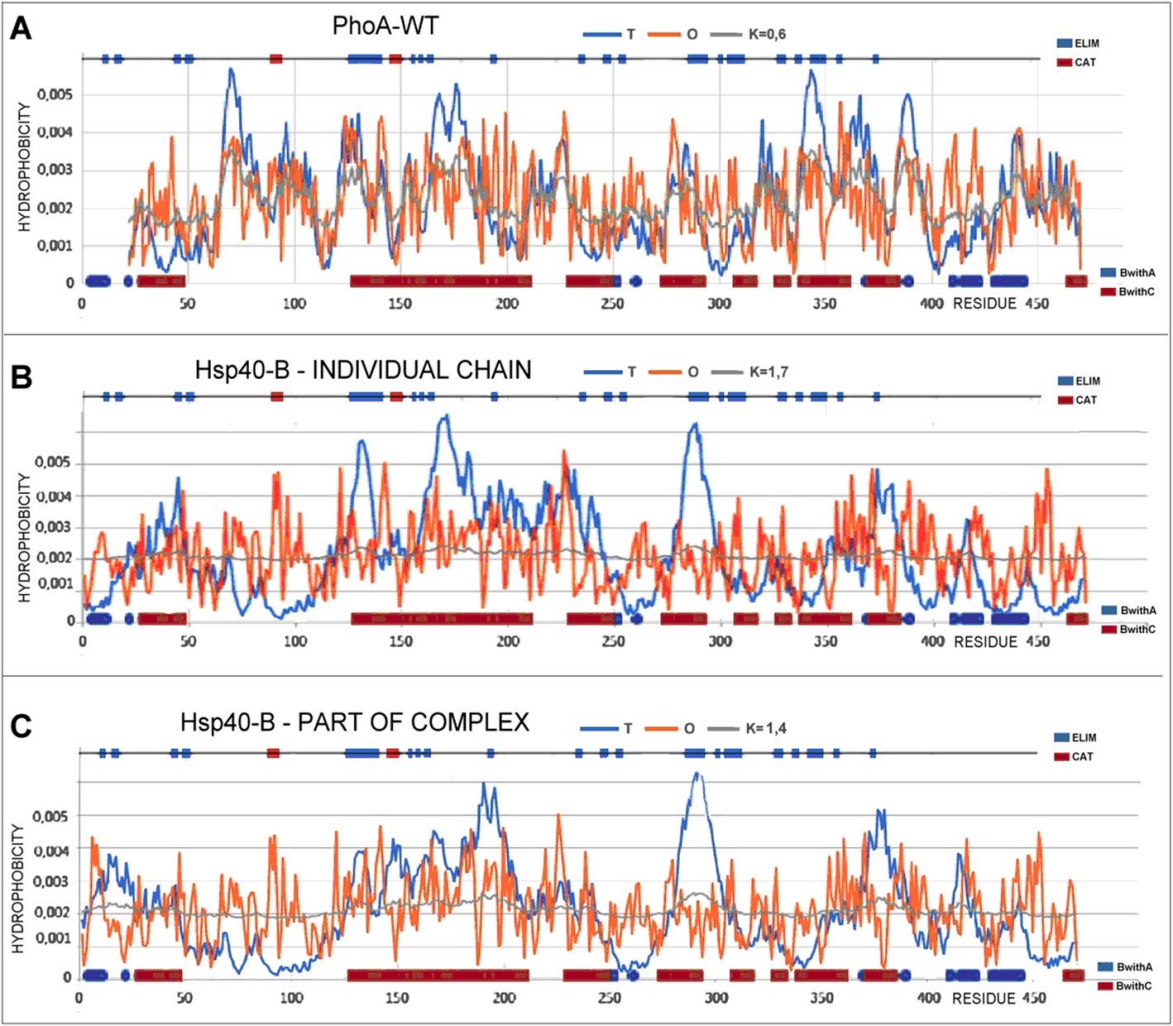
The T, O and M profiles for: **A** PhoA-WT. **B** PhoA-U (chain Hsp40-B)—as individual structural unit. **C** PhoA-U (chain Hsp40-B)—as a part of the complex Hsp40-Complex). On the x-axis the residues engaged in the interaction in PhoA-U are marked: with chain Hsp40-A (blue) and Hsp40-C (blue). Top line—red—catalytic residues, blue—residues eliminated from PhoA-WT to reach the RD < 0.5

The visualization of the RD and K parameters (Fig. 5) reveals a clearly better fit of the T and O distributions for the PhoA-WT-A form compared to the PhoA-U. The M distribution for K = 0.6 partially reproduces the T distribution. Obtaining the value RD < 0.5 requires the removal of numerous residues (showing a significant deviation of both T and O distributions), which also show a significant dispersion along the entire chain. Such a situation indicates inconsistent micelle-like protein folding.

The M distribution is important here, which for the PhoA-U form takes the form of a horizontal line, i.e. similar to the R distribution. The R distribution means complete independence from the influence of the polar water environment, introducing an almost uniform distribution of hydrophobicity along the entire chain.

**Fig. 6:**
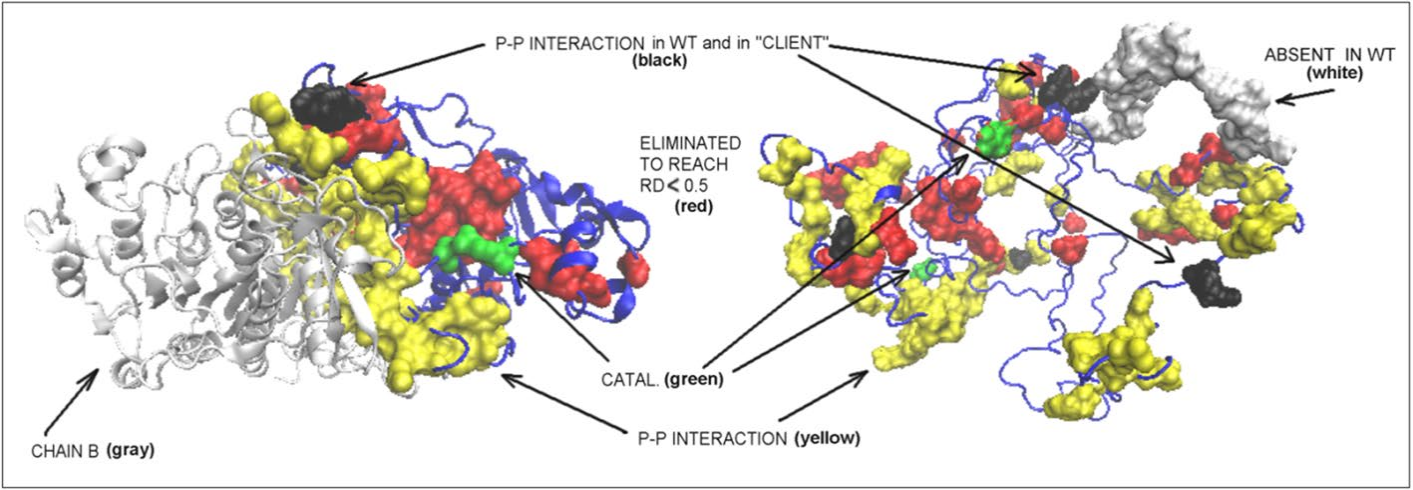
3D visualization of the PhoA-WT (left) and PhoA-U structures (right). Left—PhoA-Dimer WT—chain B—white. Right—PhoA-U – white fragment space filling—the N-terminal fragment absent in final PhoA-WT form of the discussed enzyme. Residues distinguished: red space filling—residues eliminated to reach status expressed by RD < 0.5, green – catalytic residues, yellow—residues engaged in P–P interaction in final PhoA-WT; black – residues engaged in P-P inreaction and simultaneously emgaged in P-P interaction

The discrepancies in the T and O distributions in proteins are of a different nature. There are examples of proteins with RD values > 0.5 (e.g. lysozyme [13]), where the elimination of several residues results in RD < 0.5. The common spatial location of these residues turns out to build an active center. This is not the case in the discussed PhoA-WT protein. Here, obtaining the value RD < 0.5 requires the elimination of a large number of residues located in a significant dispersion along the chain (top lines Fig. 5). This means a different folding strategy, where the chain adapts to a non-aquatic environment. The micelle-like arrangement in PhoA-WT is present to a negligible extent. This may be explained by the involvement of the chaperone in the folding of this protein. In the discussed system, the chaperone is treated as a supplier of the external force field, actively participating in the folding process by isolating the folding chain completely from the influence of the polar water environment. The localisation of chain fragments distinguished according to interaction with chaperone chains A and C are shown in Fig. 6. to trace the structural changes after final step of folding.

It is important to analyze the status of catalytic residues and their immediate surroundings. The catalytic residue 102S ± 5 (together with the immediate vicinity of ± adjacent residues in the chain sequence) shows a status described by the RD < 0.5 in all forms discussed. In the case of PhoA-U, this value is slightly above the threshold 0.5, while the residue 166R ± 5 in all forms shows a status far from micelle-like order. It can be speculated that this common status of catalytic residues is already imposed in the form of PhoA-U.

**Fig. 7:**
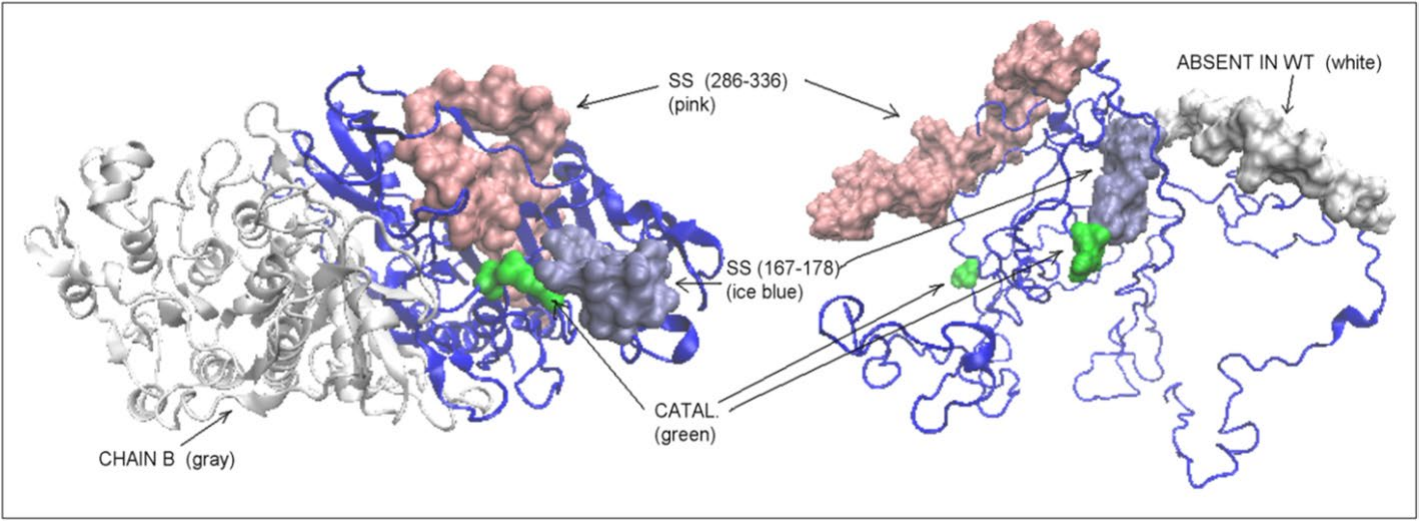
3D presentation of SS-bonds localisation in: Left – PhoA-WT form—the SS bond 167–178—ice blues space filling, SS bond 286–336—pink space filling. Chain in white—chain B in homodimer. Right—PhoA-U form with chain fragments limited by SS bonds distinguished—colours as in WT. Residues in green—catalytic residues

The presence of disulfide bonds is important for tertiary structure stabilization. The disulfide-bonded chain fragment 168–178 appears to represent a micelle-like arrangement in PhoA-WT. The SS-binding of 286–336 in the PhoA-WT-AB homodimer structure shows RD > 0.5. However, from the point of view of the structure of a single chain, its status is determined by the value of RD < 0.5. The status of both segments in the case of PhoA-U is described by RD values > 0.5 (Table 2, Fig. 7).

Residues that build the interface play a specific role in protein complexes. These residues—if the complexation is based on the hydrophobic interactions—very often show a local excess of hydrophobicity. The exposure of hydrophobicity promotes the complexation of another protein, thus disturbing the 3DGaussian distribution within the monomeric unit. In such a situation, the stepwise elimination of residues for the rest of the chain except those included in the interface results in the significant reduction of the interface’s RD value [22]. In the example discussed here, this is not the case. The increased RD value for interface building residues is observed for a single PhoA-WT chain. The remaining part of the chain (after eliminating the residues that build the interface) is described by the reduced value of RD. This means that the interface is partially encoded in the monomer structure, although the formation of a homodimer does not result in the appearance of the micelle-like arrangement for the dimer. Here one should refer to the interfaces, that only stabilize the complex. This is the case, for example, in distrophin, where the purpose of the domain with a clearly defined hydrophobic core is to stabilize the system subjected to numerous external stresses, and where the stable hydrophobic core prevents destabilization of the system [22].

**Fig. 8:**
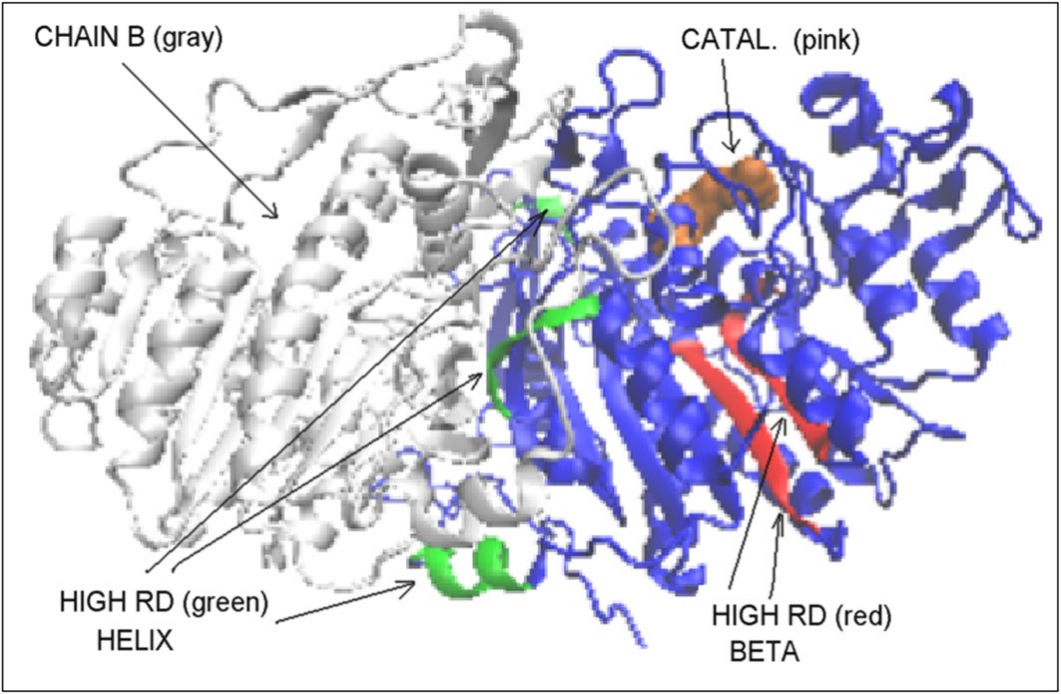
3D presentation of PhoA-WT with the residues marked as follows: Pink space filling – catalytic residues. Red—Beta-sheet fragment of significantly higher RD status with respect to PhoA-U. Green—helical fragments of significantly higher RD values in PhoA-WT form with respect to PhoA-U

The analysis of the status of individual fragments of the chain with a specific secondary structure in PhoA-WT in comparison with the form represented by these segments in PhoA-U reveals a significant increase in the RD value. Their location is shown in Fig. 8 indicating the mismatch of the centrally located part of the β-sheet.

In summary: the analysis of the structure of PhoA-WT protein chains, it should be emphasized that their status is far from micelle-like, indicating the presence of a factor significantly reducing the influence of polar water as a supplier of external force field. This is indicated by a large number of residues showing maladjustments between T and O distributions distributed along the entire chain. The inability to identify one common location of such maladjustments proves the need for the presence of an environment other than polar water in the folding process.

The status of the PhoA-U (Hsp40-B) chain shows a specific, very far from micelle-like order, of O distribution with a significantly high value of K parameter. This allows to assess the influence of the immediate environment introduced by the chaperone on the folding of the chain. The M distribution for this chain is similar to that of R one, indicating almost complete isolation from the influence of the polar water environment.

### Chaperone Hsp40 as a force field supplier in PhoA folding

The presence of the Hsp40 chaperone can be regarded as an external force field provider in the folding process of PhoA. The assessment of its impact on the basis of the FOD-M model is given in Table 3. The analysis of the status of the PhoA-U chain residues involved in the interaction with the relevant chaperone chains may answer the question about the contribution of the chaperone to the folding process. In the final structural form of PhoA-WT, the status of the sections involved in the PhoA-U structure in interaction with the chaperone (chain A and C) turns out to be far from the micelle-like status. On the other hand, the part of the chain not involved in the interaction with the chaperone chains shows the status closest to the micelle-like status (Table 3), although also at the level with RD > 0.5.

**Table 3:** The values of RD and K parameters for the part of the PhoA-WT chain involved in the interaction with the chains (A and C) in the Hsp40-Compl complex

Interaction	RD	K
With Chain A	0.638	0.8
With Chain C	0.604	0.7
Not engaged	0.584	0.5

**Fig. 9:**
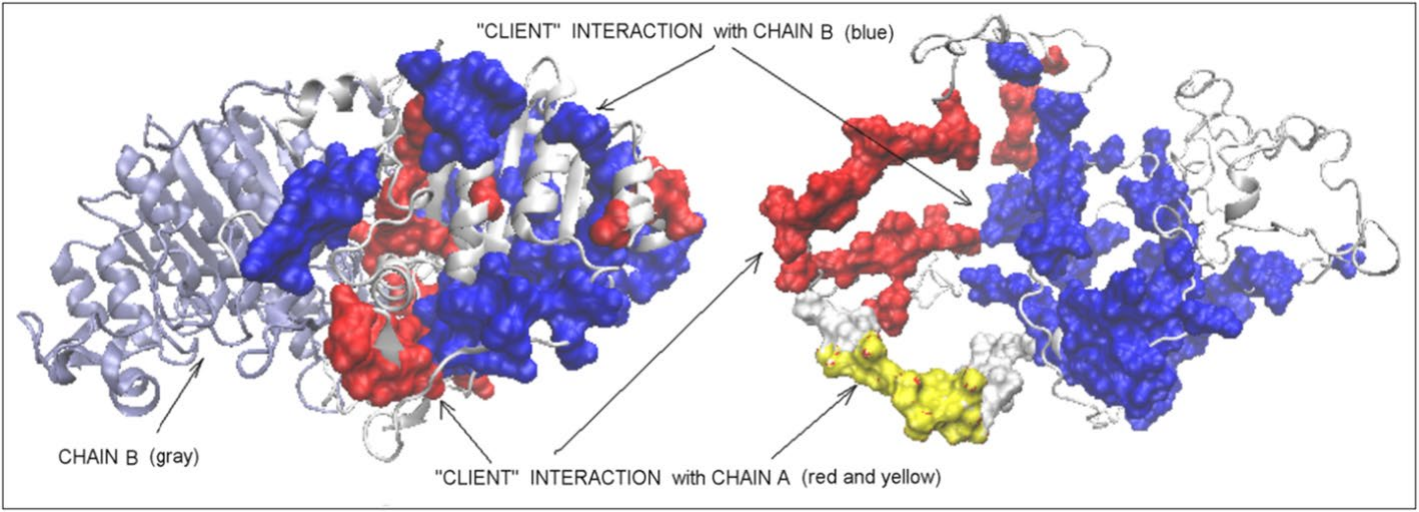
3D presentation with residues marked as follows: red—engaged in the interaction with chain A in Hsp40-Complex**,** blue –engaged in interaction with chain C in Hsp40-Complex. Left—PhoA-WT: ice blue—chain B in PhoA-WT. Right—PhoA-U: white space filling fragment—fragment absent in PhoA-WT; yellow fragment—fragment interacting with Hsp40-A in Hsp40-Complex

The location of these segments in the 3D structure of the PhoA-WT form reveals the role of the residues involved in the interaction with the Hsp40-A chain in the interchain interface area of the PhoA-AB complex. In contrast, the residues involved in the PhoA-U form in the interaction with the C chain (Hsp40-C) show surface localization in the PhoA-WT form. In other words, the chaperone determined the form of the interface and the specificity of the surface in the final form of PhoA-WT (Fig. 9.).

**Fig. 10:**
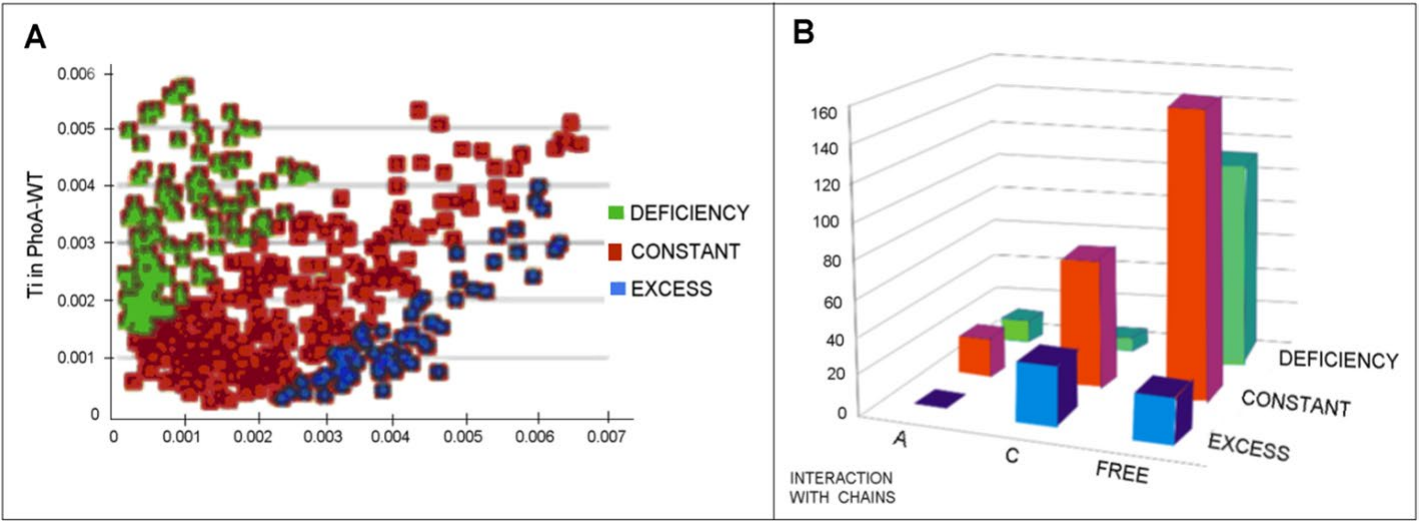
The search for factors determining the structural changes: **A** Change in the T status of the amino acids present in the structure of the enzyme in question. The Residues red—no changes in status, blue—excess in PhoA-U, green—defficiency in PhoA-U assessed versus the status in WT form. **B** The numbers of residues engaged in the interaction with Hsp40-A (left row), Hsp40-C (central row) and no interacting with Hsp40 (free—right row) respect to their belonging to areas distinguished in A. Colors as in A. Residues representing defficiency—green bars, residues representing the status accordant with T distribution in both structural forms (red bars) and residues representing excess of hydrophobicity (blue bars)

Comparing the forms of PhoA-U with PhoA-WT (Fig. 10A) one can notice a change in the T values of individual residues. Low T values indicate a surface location, far from the center. On the other hand, high T values indicate a central position. Therefore, changing the low T values in PhoA-U to high T values in PhoA-WT indicates a shift towards the center of the final structure. Figure 10B reveals that PhoA-U (and thus PhoA-WT) residues being in contact with Hsp40-C contribute the most to this rearrangement. On the other hand, the residues located on the surface in the PhoA-U that have migrated to the center are most involved in contact with the Hsp40-A chain. The residues that did not change their location in relation to the center of the molecule are mainly the residues without the contact with the chaperone chains.

The results given above allow us to speculate on the folding mechanism of the protein in question. Sections involved in interactions with A and C chains (PDB ID—6PSI) in the final structure show a status far from micelle-like organization with high RD and K values (Table 3). The structure of the relevant segments, frozen by interactions with the chaperone, enables the interaction-free part of the chain to fold according to the rules applicable in the aqueous environment, leading to a structuring similar to the micelle-like form (the lowest RD and K values for the compared sections of the PhoA-U protein chain).

### The structure of Hsp40 complex

**Fig. 11:**
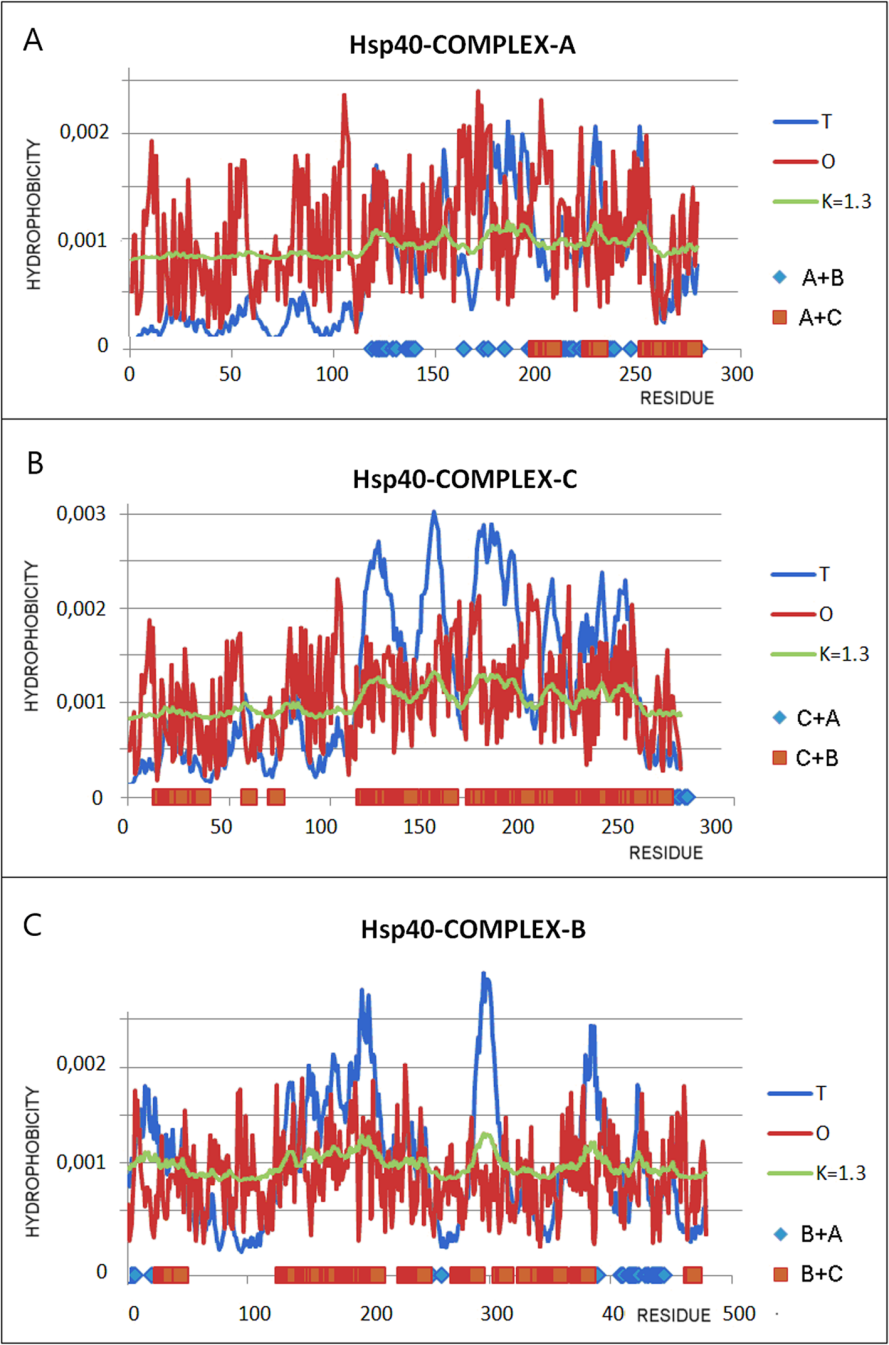
Hydrophobicity distributions: T (blue), O (red) and M (gray) for chains treated as parts of complex (3DGauss function generated for complete complex). **A** Hsp40-A. **B** Hsp40-C. **C** Hsp40-B (PhoA-U)

The use of the FOD-M model to analyze the Hsp40-Comp complex (DnaJ homodimer + client protein) reveals the high RD and K values (Table 4), and when analyzing the T, O and M profiles, significant discrepancies between the O and T distributions can be observed (Fig. 11). The M distribution obtained for a high value of K is expressed in the form of a line parallel to the x-axis, which means an approximation to the R distribution. This means an approximation of the force field devoid of the specificity of the aquatic environment. Thus, the folding process takes place in an anhydrous environment. The R distribution, which is similar to the M distributions for the entire complex, suggests that this structure is located in a kind of „water-vacuum". The presence of a polar external force field characteristic of a polar water environment is not revealed.

**Table 4:** A set of parameters describing the status of the Hsp40-Complex complex (chaperone homodimer + "client" protein)

Chains—składniki kompleksu- chain	RD	K	PP interaction RD	No P–P interaction RD
Hsp40-A, Hsp40-C, PhoA-U (chain B)	0.738	1.3	0.658	0.728
Hsp40-Dimer A + C in complex	0.738	1.3	0.682	0.743
Hsp40-A in complex	0.738	1.2	0.641	0.743
Hsp40-C in complex	0.698	0.9	0.643	0.642
PhoA–U in complex	0.749	1.4	0.651	0.766

The statuses of individual units treated as components of the complex expressed by the values of RD and K are very close to the description of the entire complex.

The highest value of RD and K, which is shown by PhoA-U treated as a component of the complex, is noteworthy. This is interpreted as imposing a structuring far removed from that which the chain obtains in an aqueous environment. Similarly, an M distribution similar to the R one reveals structuring in the „water-vacuum" (Fig. 11).

The high RD value of the interface residues (P-P and NoP-P) in the discussed complex suggests that this complex was not formed as a result of interactions directed by the aquatic environment. Using the FOD-M model, the mechanism of formation of protein complexes can be explained as an interaction of surface-exposed hydrophobic residues, which in turn gives the interface the status of a hydrophobic core component [22]. This is not the case in given example.

The status expressed by the values of RD and K (Table 4) visualizes a set of T, O and M profiles for the discussed units. The 3D Gaussian function was generated for a complex containing all three chains.

Positions on the horizontal axis—the residues involved in interactions with other chains of complex. Legends given on left site.

Looking ate Fig. 11 one can observe that the distributions O and T are significantly different, but the M distributions are comparable for All chains.

### How a chaperone chain folds?

**Fig. 12:**
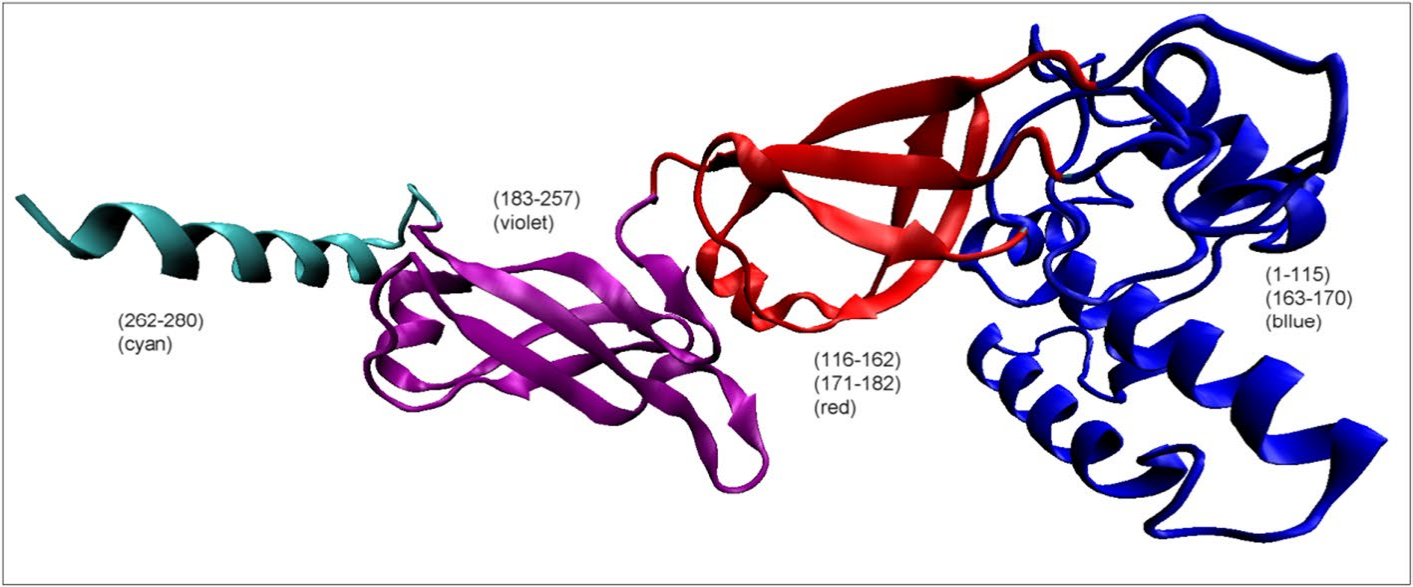
3D presentation of single Hsp40 chain with highlighted sections treated as independent structural units. (color denotations as in Table 5.). Parts denoted by red are characterized by the value of RD < 0.5

One can also ask about the way of obtaining the structure by the Hsp40-A and Hsp40-C chains. The status of these chains (dimer) treated as individual structural units is characterized by the values of RD = 0.778 and K = 1.7. So this is a status far from the form obtained spontaneously in the aquatic environment. The status of single chain Hsp40-A is described by RD = 0.646 and K = 0.8. It suggests no water-directed folding. In the structure of a single chain of the Hsp40 chaperone, the presence of domains according to the CATH criteria was not identified [24, 25]. However, for the purposes of the present work, the components of this protein structure—pseudo-domains—can be distinguished on the basis of visual analysis (Fig. 12).

**Table 5:** The values of RD and K parameters describing the status of domains distinguished in a single Hsp40 chain

Domain	Fragment	RD	K	Number of residues eliminated
1. (Blue)	1–107	0.514	0.4	4
2. (Blue)	1–115	0.499	0.4	
3. (Blue)	(1–115) + (163–170)	0.519	0.4	7
4. (Red)	(116–162) + (171–182)	0.353	0.1	
5. (Violet)	(183–257)	0.595	0.7	15
6. (Cyan)	(262–280)	0.592	0.4	3
7. Violet + Helix	(183–280)	0.563	0.5	13

Column on right—number of residues eliminated to reach RD < 0.5

The pseudo-domain status shows relatively very low K values suggesting a relatively low contribution of non-aqueous factors. RD values are slightly above the limit of RD = 0.5. The exception is the pseudo-domain 183–280. This part of the chain is involved in the interaction with the second monomer. The reason for this disorder may therefore result from the mutual influence of both chains in the interaction area. However, this complexation is also not based on hydrophobic interactions. In the structure of the dimer limited to these two pseudo-domains, there is a significant deviation from the micelle-like arrangement (RD = 0.676, for the P-P part RD = 0.561 and for the NoP-P RD = 0.682).

For the remaining pseudo-domains, however, it can be assumed that they folded based on the influence of the aquatic environment.

## Discussion

In our previous works related to protein’s structure, a hydrophobic core stabilizing the tertiary structure was repeatedly identified using the FOD-M model. This structure can be found in numerous proteins like down-hill, fast-folding, ultra-fast-folding and antifreeze type II ones [15]. The structures having a hydrophobic core can be found in the vast majority of domains identified in the PDB database [26]. This phenomenon can be interpreted as the effect of the active participation of water environment, which directs the localisation of hydrophobic residues to the center of a protein molecule with the exposure of polar ones on the surface. Very often the participation of disulfide bonds in the stabilization of protein’s tertiary structure favors the arrangement of a hydrophobic core, but it can be also the only factor responsible for its stabilization [27]. Proteins with a longer than 150 aa chain (being the limit for a domain) without a hydrophobic core are stabilized by the disulfide bonds. Undoubtedly, the presence/absence of a hydrophobic core imposes appropriate dynamics and allowable structural changes, including those related to a [22] function.

The water-based external force field can be modified by the presence of chaperones as discussed in the present work. The specificity of the hydrophobicity distribution obtained in this way relies on „imposing” uniform distribution.

To illustrate this, the reference enzyme lysozyme can be used (RD value = 0.529). Exceeding the threshold value RD = 0.5 is caused only by the 3 residues. These are the two catalytic residues 35E and 53D and the residue 128C. The location of 128C in the immediate vicinity of the substrate binding cavity should be emphasized, which gives the specificity of this site related to enzymatic activity. Eliminating these three residues results in the obtaining a status with RD < 0.5, so In this way a part of the protein was identified which, arranged according to the micelle-like scheme, ensures not only the solubility of the protein, but probably also the appropriate dynamics, including local one, related to biological activity. The folding of this protein can therefore be treated as a synergy effect of non-binding interactions and the active participation of an external force field from water [13]. Identification of the part with micelle-like order in the protein (PhoA-WT) discussed in the present work requires the elimination of numerous residues significantly distributed along the chain. [13]. This means the effect of deliberately preventing micelle-like ordering, which is ensured by the chaperone environment. Folding of the PhoA-U chain has a common feature with the process taking place in the endoplasmic reticulum, where the N-terminal fragment is rigidly anchored in the membrane [28–34]. However, the conclusions drawn here cannot be generalized due to the very diverse structures of chaperones with the folding proteins. The only thing that can be generalized is the form of the environmental imposition for the folding protein. This environment is different from the aquatic environment and can be expressed In the FOD-M model by the high RD and K values for both the folding protein and the surrounding chaperone. The role of Sect. "Introduction"-22 in the PhoA-U chain should also be emphasized, as it is almost entirely involved in the interaction with the Hsp40-Compl A chain and is removed leading to the final structure of the enzyme. This section, together with the A chain of the chaperone, is a factor referred to as a component of external conditioning, i.e. a factor modifying the external force field for the folding protein. The status of the PhoA-U chain with respect to the entire Hsp40 complex (Fig. 5B, C) shows an M distribution close to the horizontal line (almost uniform distribution). Such a system can be defined as a specific „water-vacuum" devoid of any influence of the aquatic environment (K value > 1). The status of catalytic residues seems to be already defined in the form of PhoA-U, which means its purposefulness for obtaining the final form leading to obtaining a biologically active structure.

The role of an external force field as an active participant in the folding process directly affects the aquatic environment. Changing the structure and ordering of water molecules turns out to be critical for the course of numerous reactions, including protein folding in particular [35–40], the phenomenon of accelerating the reaction on the surface [41–45], the process of wetting hydrophobic surfaces [46–49] indicated as important for the course of numerous processes. This also applies to amyloid transformation, which proceeds with an increased air–water interphase (effect of shaking) [50]. Therefore, the participation of the immediate environment as an active player in the protein folding process, the characteristics of which are expressed by the K parameter in the FOD-M model, seems to be important for the consideration of protein structuring, including amyloid transformation [51]. The discussed example of the role of a chaperone as an external force field provider is closely related to the participation of prefoldins in the process of folding supported by other proteins [52].

The model presented in this paper is oriented on In Silico structure prediction for proteins folded in assistance of chaperones or other molecules delivering specific external force field. The progress in chaperone role recognition is made in experimental as well computer-based techniques.

Recently proposed models like Wako-Saitô-Muñoz-Eaton (WSME) model succesfully predicts the structure of domains [53, 54]. However, according to FOD-M-based analysis, domains are mostly characterised by very low K parameter [26]. One-state model for folding process simulation is discussed in context of cooperativity, which appears to be difficult for simulation-based techniques [55]. Significant progres in experimental observations of chaperone-assisted folding espetially in context of refolding process aimed on structural corrections occurring in folding process is discussed in [56]. Highly complex system of protein quality control systems called also chaperone-assisted selective autophagy (CASA) responsible for proteinostatis appears to be to complicated to be simulated In Silico [57]. Despite of high complexity of chaperone construction the dynamics simulation of plasmodial HSP70 and HSP110 to reveal the disfferences in respect to human forms of these proteins in comparison to their human orthologs [58]. The complexity of the system is even higher taking into account the necessary co-operation of chaperones with co-chaperones, as it is shown in p50/Cdc37 and Hsp90 partnership in the regulation of protein kinanses [59].

Experimental examination of the mutation reveals the influence co-chaperone regulation in folding process [60].

Mechanism of cellular chaperone machinery involved in Rubisco biosynthesis is shown as engaging the DnaK and DnaJ from Hsp70/40 [61].

The participation of Hsp90 and Hsp70 chaperone families on human metabolic enzymes espetially their participation in enzymatic activities as participating in translocation and degradation of methabolic enzymes highlights their participation in disease-related disorders [62].

The protein-binding site in relation to water-exposed surface of chaperone Hsp70 discussed in context of aggregation devoiding is shown on the binding sites of 2258 *Escherichia coli (E. coli)* proteins [63].

Experimental examination of chaperone activity on cellular level under changed conditions (extremely-lowfrequency electromagnetic fields (ELF-EMF)) was examined to reveal the time of 3 h activation and 12 h return to basal level as potential medical treatemnt in selected diseases [64].

Hsp70 is also interpreted as the tool delivering nascent membrane proteins of appropriate structure for that environemnt [65].

This short revision of the recent progres in the chaperon-assisted protein folding introduces only to egerly developed activity in recognition of chaperone activity.

The self-organized polymer (SOP) model applies the coarse-grained representation of protein structure to simulate the many-steps process of folding and unfolding including forced forms of these processes. The unfolding process as shown in [66] is the effect of changes of internal interaction (internal force field). Similar effect can be reached according to FOD-M model defining the external force field with higher K value in comparison with the value describing the native structure. Step-wise enlargement of the size of appropriate 3DGauss (or form representing M distribution – Eq. 6) directs the folding process (during the FOD-M-based optimisation procedure, which shall follow internal-force field optimisation procedure) toward the more and more extended structural form. Any form of internal force field (including coarse-grained representation) may be applied in such procedure. The UNRES force field applied for folding simulation in the prresence of external force field represents also the coarse-grained force field [67].

## Conclusions

The use of the FOD-M model allows for a quantitative assessment of the change in the folding environment in relation to the aqueous environment, thus determining the role of the chaperone as the supplier of the external force field for the protein folding process. The tertiary structure of PhoA-WT is stabilized by disulfide bonds in the absence of a hydrophobic core. The mismatch of the hydrophobicity distribution towards the micelle-like system applies to the entire chain (as opposed to the proteins, where the few residues showing such a mismatch are located in a 3D structure in a common place, often creating a precisely defined active center). This means that the uniform distribution of hydrophobicity is deliberate and is obtained by the environment of the external force field created in the discussed example by the chaperone. The chaperone’s environment can be defined as a kind of a „water-vacuum" enabling the distribution of hydrophobicity in accordance with the uniform distribution.

The introduction of an external M-distributed field to the in Silico simulation of the protein folding process should direct this process towards the presence of the chaperone. The current work is another example of treating the presence of a factor other than the aqueous environment (membrane [15], prefoldin [52]) as a form of external force field actively participating in the protein folding process.

The generated structure of the PhoA-WT enzyme formed as a result of the external force field provided by the chaperone, in turn, creates an external field for the catalysis process. The characteristics of this field can be determined by the parameter K, which specifies the degree of change to the aqueous environment directing the folding process towards the construction of a central hydrophobic core.

The FOD-M model treats the distribution of hydrophobicity (internal hydrophobic force field) as the effect of external force field (chaperone) influencing the folding proces. This is why the M function (3DGauss function modified by K parameter) is assumed to be the definition of the external force field for prtein folding simulation in **Ab initio** model. The folding simulation controled by the energy (non-bonding interaction) optimisation shall also take the external force field inluence as optimisation factor under consideration. The simulation shall follow the step-wise adaptation to the directing role of environemntal influence. The **Ab Initio** folding simulation of alkaline phosphatase folding shall be posssible in the presence of external force field expressed with K = 0.6 modification of 3DGauss function.

## Data Availability

All data can be available on request addressed to the corresponding author. The program allowing calculation of RD is accessible on GitHub platform: https://github.com/KatarzynaStapor/FODmodel and on the platform https://hphob.sano.science.

## References

[1] Hartl FU (1995). Principles of chaperone-mediated protein folding. Philos Trans R Soc Lond B Biol Sci.

[2] Jiang Y, Rossi P, Kalodimos CG (2019). Structural basis for client recognition and activity of Hsp40 chaperones. Science.

[3] Kampinga HH, Craig EA (2010). The HSP70 chaperone machinery: J proteins as drivers of functional specificity. Nat Rev Mol Cell Biol.

[4] Zhu M, Ou D, Khan MH, Zhao S, Zhu Z, Niu L (2020). Structural insights into the formation of oligomeric state by a type I Hsp40 chaperone. Biochimie.

[5] Saibil H (2013). Chaperone machines for protein folding, unfolding and disaggregation. Nat Rev Mol Cell Biol.

[6] Lee S, Fan CY, Younger JM, Ren H, Cyr DM (2002). Identification of essential residues in the type II Hsp40 Sis1 that function in polypeptide binding. J Biol Chem.

[7] Borges JC, Seraphim TV, Mokry DZ, Almeida FC, Cyr DM, Ramos CH (2012). Identification of regions involved in substrate binding and dimer stabilization within the central domains of yeast Hsp40 Sis1. PLoS ONE.

[8] Faust O, Rosenzweig R (2020). Structural and Biochemical Properties of Hsp40/Hsp70 Chaperone System. Adv Exp Med Biol.

[9] Alderson TR, Kim JH, Markley JL (2016). Dynamical Structures of Hsp70 and Hsp70-Hsp40 complexes. Structure.

[10] Cyr DM, Ramos CH (2015). Specification of Hsp70 function by Type I and Type II Hsp40. Subcell Biochem.

[11] 6PSI Jiang Y, Rossi P, Kalodimos CG. Structural basis for client recognition and activity of Hsp40 chaperones. Science 2019; 365(6459):1313–1319. 10.1126/science.aax1280. 10.1126/science.aax1280 PMC7023980 31604242

[12] 1EW81EW8Holtz KM, Stec B, Myers JK, Antonelli SM, Widlanski TS, Kantrowitz ER. Alternate modes of binding in two crystal structures of alkaline phosphatase-inhibitor complexes. Protein Sci 2000; 9(5): 907–15. 10.1110/ps.9.5.907. 10.1110/ps.9.5.907 PMC2144633 10850800

[13] Roterman I, Konieczny L. Protein Is an Intelligent Micelle Entropy, 2023, *25*(6), 850; 10.3390/e25060850 10.3390/e25060850 PMC10297380 37372194

[14] Konieczny L, Brylinski M, Roterman I (2006). Gauss-function-Based model of hydrophobicity density in proteins. In Silico Biol.

[15] Banach M, Stapor K, Konieczny L, Fabian P, Roterman I (2020). Downhill, ultrafast and fast folding proteins revised. Int J Mol Sci.

[16] https://www.ks.uiuc.edu/Research/vmd/. Accessed June 2023

[17] Humphrey W, Dalke A, Schulten K (1996). VMD: visual molecular dynamics. J Molec Graphics.

[18] Levitt M (1976). A simplified representation of protein conformations for rapid simulation of protein folding. J Mol Biol.

[19] Kullback S, Leibler RA. On information and sufficiency. Ann Math Stat. 1951, 22(1), 79e86. 10.1214/aoms/1177729694.

[20] Roterman I, Stapor K, Gądek K, Gubała T, Nowakowski P, Fabian P, Konieczny L (2022). Dependence of protein structure on environment: FOD model applied to membrane proteins. Membranes.

[21] Roterman I, Stapor K, Fabian P, Konieczny L (2021). The functional significance of hydrophobic residue distribution in bacterial beta-barrel transmembrane proteins. Membranes.

[22] Dygut J, Kalinowska B, Banach M, Piwowar M, Konieczny L, Roterman I (2016). Structural interface forms and their involvement in stabilization of multidomain proteins or protein complexes. Int J Mol Sci.

[23] Roterman I, Konieczny L, Stapor K, Slupina M. Force field for enzymatic catalysis. Submitted.

[24] CATH https://www.cathdb.info/. Accessed June 2023.

[25] Sillitoe I, Bordin N, Dawson N, Waman VP, Ashford P, Scholes HM, Pang CSM, Woodridge L, Rauer C, Sen N, Abbasian M, Le Cornu S, Lam SD, Berka K, Varekova IH, Svobodova R, Lees J, Orengo CA (2021). CATH: increased structural coverage of functional space. Nucleic Acids Res.

[26] Sałapa K, Kalinowska B, Jadczyk T, Roterman I (2012). Measurement of hydrophobicity distribution in proteins - non-redundant protein data bank. Bio-Algorithms Med-Syst..

[27] Banach M, Kalinowska B, Konieczny L, Roterman I (2016). Role of disulfide bonds in stabilizing the conformation of selected enzymes: an approach based on divergence entropy applied to the structure of hydrophobic core in proteins. Entropy.

[28] Coleman OI, Haller D (2019). ER Stress and the UPR in shaping intestinal tissue homeostasis and immunity. Front Immunol.

[29] Rutkowski DT, Kauffman RJ (2004). A trip to the ER: coping with stress. Trends Cell Biol.

[30] Johannes L, Popoff V. Tracing the retrograde route in protein trafficking. Cell. 2008;135(7);1175–87. 10.1016/j.cell.2008.12.009. 10.1016/j.cell.2008.12.009 19109890

[31] Rahman S, Jan AT, Ayyagari A, Kim J, Kim J, Minakshi R (2017). Entanglement of UPR^ER^ in aging driven neurodegenerative diseases. Front Aging Neurosci.

[32] Martinez-Vicente M, Sovak G, Cuervo AM (2005). Protein degradation and aging. Exp Gerontom.

[33] Zhou AX, Tabas I (2013). The UPR in atherosclerosis. Semin Immunopathol.

[34] Chitnis N, Pytel D, Diehl JA (2013). UPR-inducible miRNAs contribute to stressful situations. Trends Biochem Sci.

[35] Romero-Montalvo E, DiLabio GA (2021). Computational study of hydrogen bond interactions in water cluster-organic molecule complexes. J Phys Chem A.

[36] Hazra S, Kaur G, Handa S (2021). Reactivity of styrenes in micelles: safe, selective, and sustainable functionalization with azides and carboxylic acids. ACS Sustain Chem Eng..

[37] Nguyen D, Casillas S, Vang H, Garcia A, Mizuno H, Riffe EJ, Saykally RJ, Nguyen SC. Catalytic mechanism of interfacial water in the cycloaddition of 38. quadricyclane and diethyl azodicarboxylate. J Phys Chem Lett. 2021;12(12), 3026–3030. 10.1021/acs.jpclett.1c00565 10.1021/acs.jpclett.1c00565 33734703

[38] Sarathkumar S, Kavala V, Yao C-F (2021). A water-soluble rhenium(I) catalyst for the regio- and stereoselective C(sp2)–H alkenylation of N-pyridyl-/N-pyrimidylindole and the N–H alkenylation of N-pyrimidylaniline derivatives with Ynamides. Org Lett.

[39] Duong U, Ansari TN, Parmar S, Sharma S, Kozlowski PM, Jasinski JB, Plummer S, Gallou F, Handa S (2021). Nanochannels in photoactive polymeric Cu(I) compatible for efficient micellar catalysis: sustainable aerobic oxidations of alcohols in water. ACS Sustain Chem Eng.

[40] Huang J, Chen W, Liang J, Yang Q, Fan Y, Chen M-W, Peng Y (2021). α-Keto acids as triggers and partners for the synthesis of quinazolinones, quinoxalinones, benzooxazinones, and benzothiazoles in water. J Org Chem.

[41] Eremin DB, Fokin VV (2021). On-water selectivity switch in microdroplets in the 1,2,3-triazole synthesis from bromoethenesulfonyl fluoride. J Am Chem Soc.

[42] Kaplaneris N, Vilches-Herrera M, Wu J, Ackermann L (2022). Sustainable ruthenium(II)-catalyzed C–H activations in and on H_2_O. ACS Sustain Chem Eng..

[43] Ishiyama T, Tahara T, Morita A (2022). Why the photochemical reaction of phenol becomes ultrafast at the air-water interface: the effect of surface hydration. J Am Chem Soc.

[44] Gupta A, Vankar JK, Jadav JP, Gururaja GN (2022). Water mediated direct thioamidation of aldehydes at room temperature. J Organic Chem.

[45] Cannalire R, Santoro F, Russo C, Graziani G, Tron GC, Carotenuto A, Brancaccio D, Giustiniano M (2022). Photomicellar catalyzed synthesis of amides from isocyanides: optimization, scope, and nmr studies of photocatalyst/surfactant interactions. ACS Organic Inorganic Au.

[46] Zhao S, Li K, Sun X, Zha Z, Wang Z (2022). Copper-catalyzed stereoselective [4 + 2] cycloaddition of β, γ-unsaturated α-Keto esters and 2-vinylpyrroles in water. Org Lett.

[47] Li Z, Fu C-F, Chen Z, Tong T, Hu J, Yang J, Tian SX (2022). Electron-induced synthesis of dimethyl ether in the liquid-vapor interface of methanol. J Phys Chem Lett.

[48] Sugimoto Y (2022). Seeing how ice breaks the rule. Science.

[49] Tian Y, Hong J, Cao D, You S, Song Y, Cheng B, Wang Z, Guan D, Liu X, Zhao Z, Li X-Z, Xu L-M, Guo J, Chen J, Wang E-G, Jiang Y (2022). Visualizing Eigen/Zundel cations and their interconversion in monolayer water on metal surfaces. Science.

[50] Ladner-Keay CL, Griffith BJ, Wishart DS. Shaking alone induces de novo conversion of recombinant prion proteins to β-sheet rich oligomers and fibrils. PLoS One. 2014 ;9(6):e98753. 10.1371/journal.pone.0098753. eCollection 2014. 10.1371/journal.pone.0098753 PMC4043794 24892647

[51] Roterman I, Stapor K, Fabian P, Konieczny L (2021). In silico modeling of the influence of environment on amyloid folding using FOD-M Model. Int J Mol Sci.

[52] Roterman I, Stapor K, Konieczny L. Model of external force field for protein folding proces—role of prefoldin. Submitted 10.3389/fchem.2024.1342434 PMC11002104 38595701

[53] Ooka K, Liu R, Arai M (2022). The Wako-Saitô-Muñoz-Eaton model for predicting protein folding and dynamics. Molecules.

[54] Bruscolini P, Pelizzola A (2002). Exact solution of the Muñoz-Eaton model for protein folding. Phys Rev Lett.

[55] Muñoz V, Campos LA, Sadqi M (2016). Limited cooperativity in protein folding. Curr Opin Struct Biol.

[56] Marzano NR, Paudel BP, van Oijen AM, Ecroyd H. Real-time single-molecule observation of chaperone-assisted protein folding. Sci Adv. 2022; 8(50):eadd0922. 10.1126/sciadv.add0922. 10.1126/sciadv.add0922 PMC9750156 36516244

[57] Tedesco B, Vendredy L, Timmerman V, Poletti A (2023). The chaperone-assisted selective autophagy complex dynamics and dysfunctions. Autophagy.

[58] Tripathi A, Del Galdo S, Chandramouli B, Kumar N (2023). Distinct dynamical features of plasmodial and human HSP70-HSP110 highlight the divergence in their chaperone-assisted protein folding. Biochim Biophys Acta Proteins Proteom.

[59] Prince TL, Lang BJ, Okusha Y, Eguchi T, Calderwood SK (2023). Cdc37 as a Co-chaperone to Hsp90. Subcell Biochem.

[60] Mercier R, Yama D, LaPointe P, Johnson JL (2023). Hsp90 mutants with distinct defects provide novel insights into cochaperone regulation of the folding cycle. PLoS Genet.

[61] Rydzy M, Kolesiński P, Szczepaniak A, Grzyb J. DnaK and DnaJ proteins from Hsp70/40 family are involved in Rubisco biosynthesis in *Synechocystis* sp. PCC6803 and sustain the enzyme assembly in a heterologous system. BMC Plant Biol 2023; 23(1):109. 10.1186/s12870-023-04121-1. 10.1186/s12870-023-04121-1 PMC9948308 36814186

[62] Binder MJ, Pedley AM (2023). The roles of molecular chaperones in regulating cell metabolism. FEBS Lett.

[63] Xi Chen X, Rachel B Hutchinson RB, Silvia Cavagnero S. Distribution and solvent exposure of Hsp70 chaperone binding sites across the *Escherichia coli* proteome. Proteins 2023 ; 91(5):665–78. 10.1002/prot.26456. 10.1002/prot.26456 PMC10073276 36539330

[64] Huang Z, Ito M, Zhang S, Toda T, Takeda J-I, Ogi T, Ohno K (2023). Extremely low-frequency electromagnetic field induces acetylation of heat shock proteins and enhances protein folding. Ecotoxicol Environ Saf.

[65] Shan S-O (2023). Role of Hsp70 in post-translational protein targeting: tail-anchored membrane proteins and beyond. Int J Mol Sci.

[66] Hyeon C, Dima RI, Thirumalai D. Pathways and kinetic barriers in mechanical unfolding and refolding of RNA and proteins. Structure 2006, 14(11):1633–45 10.1016/j.str.2006.09.002 17098189

[67] Roterman I, Sieradzan A, Stapor K, Fabian P, Wesołowski P, Konieczny L (2022). On the need to introduce environmental characteristics in ab initio protein structure prediction using a coarse-grained UNRES force field. J Mol Graph Model.

